# ARE POLICY AND PRACTICE CHANGE TO SUPPORT FAMILY CAREGIVERS ON THE RISE?

**DOI:** 10.1093/geroni/igz038.2741

**Published:** 2019-11-08

**Authors:** Lynn F Feinberg

**Affiliations:** AARP Public Policy Institute, Washington, District of Columbia, United States

## Abstract

The challenges and pressures of family caregiving for older relatives or friends are a reality of daily life. Better recognition of and support for family caregivers has emerged as a national health, economic and social priority that can no longer be ignored. Unlike previous generations, most (60%) family caregivers work at a paying job today in addition to caring for older relatives with a serious health condition or disability. Building a better system of care for older adults means changes in workplaces as well as health care and LTSS settings. This paper presents an analysis of the key challenges that family caregivers face, and highlights policy developments and practice change at the federal and state levels since 2015. The presentation addresses the importance of person- and family-centered care in everyday practice, and discusses the road ahead to change the culture of care.

